# *HFE* p.C282Y Polymorphism and Risk of Metabolic Syndrome Components: Systematic Review and Meta-Analysis

**DOI:** 10.3390/medicina62030589

**Published:** 2026-03-20

**Authors:** Dana Kaldarkhan, Gulnaz Nuskabayeva, Nursultan Nurdinov, Yerbolat Saruarov, Ugilzhan Tatykayeva, Ainash Oshibayeva, Karlygash Sadykova

**Affiliations:** Faculty of Medicine, Khoja Akhmet Yassawi International Kazakh-Turkish University, Turkestan 160000, Kazakhstan; dana.kaldarkhan2024@ayu.edu.kz (D.K.);

**Keywords:** metabolic syndrome, hemochromatosis, diabetes, hypertension, abdominal obesity, high-density lipoprotein cholesterol, triglycerides

## Abstract

*Background and Objectives*: Metabolic syndrome is a common condition associated with a higher risk of diabetes and cardiovascular disease. Altered iron metabolism, especially iron overload, may play a role in the development of insulin resistance, hypertension, and other metabolic abnormalities. Although the p.C282Y polymorphism of the *HFE* gene affects iron regulation and may contribute to these metabolic changes, previous studies have reported inconsistent findings, thus highlighting the need for a comprehensive meta-analysis. *Materials and Methods*: A systematic literature search was performed in PubMed, Scopus, and Web of Science to examine the associations between the *HFE* p.C282Y polymorphism and components of metabolic syndrome. Observational studies were included if they compared the frequencies of diabetes, hypertension, and abdominal obesity, as well as levels of triglycerides and high-density lipoprotein cholesterol, between carriers and non-carriers of the p.C282Y variant. *Results*: A total of 17 studies were included in the meta-analysis, and the pooled analysis demonstrated no significant association between the *HFE* p.C282Y polymorphism and diabetes, hypertension, triglyceride levels, or HDL cholesterol levels under the codominant model. Similarly, analyses performed using the homozygous model did not reveal any statistically significant associations. *Conclusions*: This meta-analysis found no statistically significant association between the *HFE* p.C282Y polymorphism and any of the considered components of metabolic syndrome under the examined genetic models.

## 1. Introduction

Metabolic syndrome (MetS) is a complex clinical phenotype defined by abdominal obesity, elevated blood pressure, dyslipidemia (characterized by high triglycerides and/or low high-density lipoprotein (HDL) cholesterol), and diabetes. These components cluster together more frequently than would be expected by chance and confer substantially increased risk for atherosclerotic cardiovascular disease (ASCVD), type 2 diabetes mellitus, and other adverse health outcomes [1,2]. This syndrome is recognized as a major public health challenge due to its rising prevalence and associated morbidity and mortality [1,3]. Although understanding the genetic basis of MetS is critical for tailored medical approaches, the condition’s polygenic nature complicates the identification of individual genes.

MetS is strongly associated with hyperferritinemia, a laboratory finding characterized by elevated serum ferritin levels. Genetic variants affecting iron homeostasis, as well as environmental and dietary factors, contribute to iron accumulation in patients with MetS, and this iron overload is implicated in the pathogenesis of insulin resistance and organ damage [4,5,6]. The associations between hyperferritinemia and metabolic dysfunction have been proven in numerous studies; for example, recent studies have reported the importance of genetic variants that regulate iron homeostasis and iron levels in tissue with respect to the occurrence and progression of diabetes and hypertension [7,8]. The accumulation of iron in the body may be associated with insulin resistance and the pathogenesis of associated organ damage. Therefore, genes related to iron metabolism disorders can act as genetic determinants for the development of diabetes, hypertension, and obesity [5].

Although iron homeostasis is largely regulated by hepcidin—a hormone whose expression is influenced by the *HFE* protein within the iron-sensing pathway—hepcidin regulation is multifactorial and involves several additional molecular and signaling mechanisms [9]. The p.C282Y mutation in the *HFE* gene is the principal genetic variant associated with hereditary hemochromatosis, an autosomal recessive disorder characterized by increased intestinal iron absorption and progressive iron overload [10,11]. Despite the fact that hundreds of studies focused on genetic associations have been published to date, the results regarding the roles of specific variants in the development of diabetes, hypertension, and obesity remain contradictory, making it difficult to understand the genetic architecture of MetS. Notably, this may be due to the small sample size in most studies [12]. Mechanistically, iron overload impairs insulin secretion through direct injury to pancreatic β-cells and promotes insulin resistance, with effects observed across the spectrum from hereditary hemochromatosis to dysmetabolic iron overload in metabolic syndrome [13,14]. According to a 2024 study, patients homozygous for the p.C282Y variant with normal serum ferritin levels showed an association between transferrin-related parameters and an increased risk of developing diabetes mellitus. Carriers of the *HFE* p.C282Y variant have also been reported to have a higher risk of diabetes mellitus and liver diseases, whereas no consistent association with hypertension was observed [15]. In parallel, Jaakko’s study suggested that hepcidin dysregulation resulting from *HFE* gene mutations may exert an inhibitory effect on renin, potentially influencing blood pressure regulation [12]. Overall, the available literature does not demonstrate a consistent association between the p.C282Y mutation and other components of metabolic syndrome, such as hypertension or dyslipidemia. Thus, while this mutation appears to be associated with an increased risk of diabetes mellitus, its relationships with central adiposity and other metabolic syndrome features remain less clearly defined. Therefore, this study examined *HFE* gene variants, focusing on the p.C282Y polymorphism and its homozygous and heterozygous genotypes; conducted a systematic review and meta-analysis regarding its associations with diabetes, hypertension, and HDL and triglyceride levels; and consequently assessed the risk of developing each component of metabolic syndrome.

## 2. Materials and Methods

### 2.1. Literature Search

We conducted a comprehensive search of scientific online databases, including Scopus, PubMed, and Web of Science, to identify articles published in English focused on carriers of the p.C282Y allele and metabolic syndrome components. We screened for articles from the establishment of each database until 30 November 2025.

The search approach was structured as follows: Diabetes: “C282Y rs1800562” AND “polymorphism” OR “variant” OR “genotype” AND “T2DM” OR “type 2 diabetes” OR “diabetes mellitus type 2” OR “diabetic condition” OR “diabetic.” Hypertension: “C282Y rs1800562” AND “polymorphism” OR “SNP” OR “genotype” AND “hypertension’’ OR “hypertensions” OR “blood pressure”. Low high-density lipoprotein (HDL): “C282Y rs1800562” AND “SNP” OR “variant” OR “genotype” AND “HDL-C” OR “high-density lipoprotein.” Hypertriglyceridemia: “C282Y rs1800562” AND “SNP” OR “variant” OR “genotype” AND “hypertriglyceridemia” OR “triglyceride.”

Additionally, we conducted a manual search of the reference lists of the included studies to identify any further potentially relevant publications. The current investigation was performed following the Preferred Reporting Items for Systematic Reviews and Meta-Analyses (PRISMA statement) guidelines (see Supplementary Table S1). The protocol of this systematic review was officially registered in the International Prospective Register of Systematic Reviews (PROSPERO), with record number CRD420261282222. The full protocol is publicly available at https://www.crd.york.ac.uk/PROSPERO/view/CRD420261282222 (accessed 05 on March 2026). No amendments were made after registration.

### 2.2. Inclusion Criteria

Two authors separately evaluated the retrieved literature for inclusion. The following inclusion criteria were applied: (1) evaluation of the p.C282Y allele rs1800562 locus polymorphism and its associated risk components of MetS, (2) availability of sufficient raw data to enable the calculation of odds ratios (ORs), and (3) provision of information on the total sample size and the number of cases.

### 2.3. Exclusion Criteria

Studies were excluded based on the following criteria: (1) meta-analysis or review, (2) duplicate material, (3) animal research, and (4) lacking relevant data.

### 2.4. Data Extraction

From each eligible study, we collected the following data: (1) first author, (2) year of publication, (3) race, (4) source of controls, (5) number of cases and controls, (6) Hardy–Weinberg, (7) adjustment factors, and (8) genotyping methods. Two investigators independently extracted the data, and any disagreements were resolved through discussion.

### 2.5. Quality Assessment of the Studies

The methodological quality and risk of bias of the included studies were independently assessed by two reviewers using validated instruments developed by the National Institutes of Health (NIH). Observational cohort and cross-sectional studies were evaluated using the NIH Quality Assessment Tool for Observational Cohort and Cross-Sectional Studies, while case–control studies were assessed using the NIH Quality Assessment Tool for Case–Control Studies. Any discrepancies between the reviewers were resolved through discussion to reach a consensus.

### 2.6. Statistical Analyses

All data analyses were performed using the Review Manager (RevMan) software (version 5.4; The Cochrane Collaboration, Copenhagen, Denmark) and Jamovi software (version 2.3.28; The Jamovi Project, Sydney, Australia). In the present study, three outcomes—diabetes mellitus, hypertension, and abdominal obesity—were treated as dichotomous variables. Pooled associations for these outcomes were estimated using a random effects model, and effect sizes are expressed as odds ratios (ORs) with corresponding 95% confidence intervals (CIs). HDL cholesterol and triglycerides were analyzed as continuous variables, as most included studies reported lipid levels as mean ± SD without categorical MetS thresholds. All lipid values were harmonized and expressed in mmol/L to ensure comparability across studies. Comparisons were performed between carriers and non-carriers of the investigated genetic variants using a random effects model. Statistical heterogeneity between the included studies was assessed using the I^2^ statistic. Values of 25%, 50%, and 75% were considered to indicate low, moderate, and high heterogeneity, respectively.

## 3. Results

### 3.1. Characteristics and Quality Assessment of the Eligible Studies

**Figure 1:**
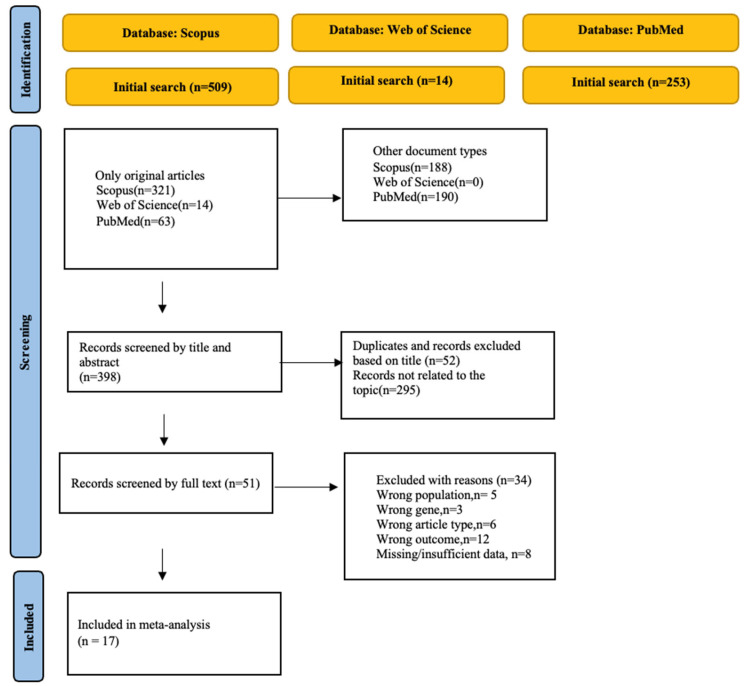
PRISMA flow chart.

Overall, 776 eligible articles were identified through screening of various databases. After screening, 17 qualified articles published between 2001 and 2024 were selected for our meta-analysis. The entire process of article selection is illustrated in Figure 1. Regarding race, all 17 were conducted among Caucasians. Genotyping was conducted using TaqMan assays, KASP genotyping technology, and conventional PCR-based approaches. Regarding the source of control subjects, 13 studies included controls from the general population, while 4 included controls from the hospital itself. The characteristics of the qualified studies are summarized in Table 1. The search strategies for the databases are presented in Supplementary Table S2.

#### 3.1.1. Association Between the p.C282Y rs1800562 Polymorphism and the Risk of Diabetes

**Figure 2:**
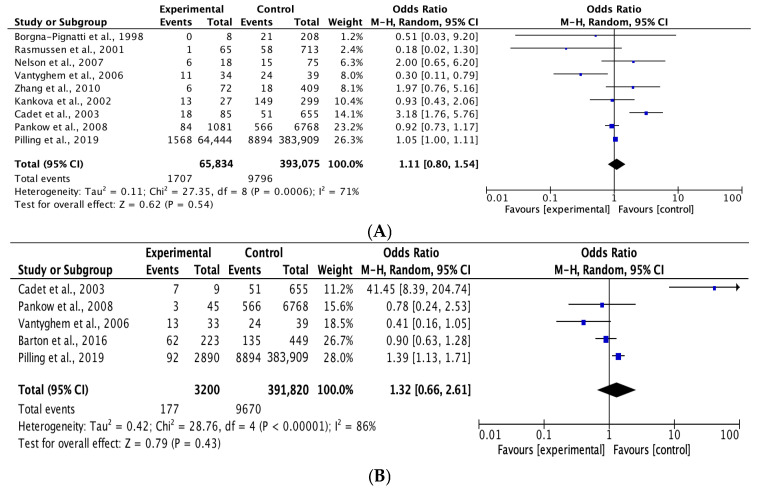
Forest plot of the sensitivity of diabetes between wild-type and p.C282Y heterozygous carriers (**A**). Forest plot of the sensitivity of diabetes between wild-type and p.C282Y homozygous carriers (**B**).

Based on the random effects model (Figure 2), the p.C282Y rs1800562 polymorphism was not associated with the risk of diabetes (p.C282Y heterozygous genotype model CY vs. CC: OR = 1.11, 95% CI = 0.80–1.54, *p* = 0.54; homozygous genotype model YY vs. CC: OR = 1.32, 95% CI = 0.66–2.61, *p* = 0.43).

#### 3.1.2. Association Between the p.C282Y rs1800562 Polymorphism and the Risk of Hypertension

**Figure 3:**
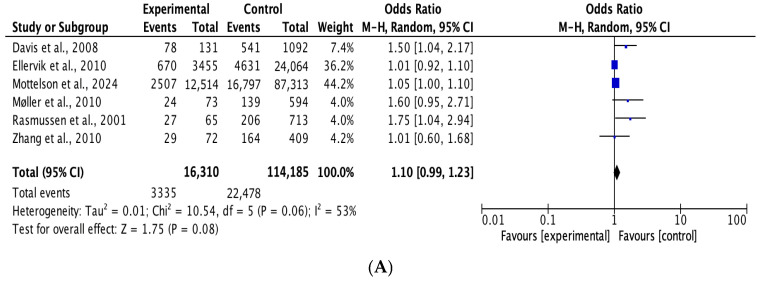

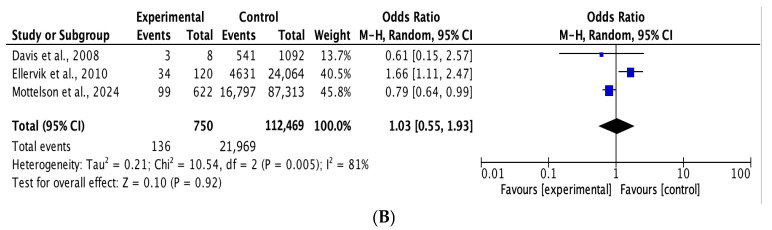
Forest plot of the relationship of hypertension between wild-type and p.C282Y heterozygous carriers (**A**). Forest plot of the relationship of hypertension between wild-type and p.C282Y homozygous carriers (**B**).

Based on the random effects model (Figure 3), the p.C282Y rs1800562 heterozygous model was not associated with the risk of hypertension (p.C282Y heterozygous genotype model CY vs. CC: OR = 1.10, 95% CI = 0.99–1.23, *p* = 0.08; homozygous genotype model YY vs. CC: OR = 1.03, 95% CI = 0.55–1.93, *p* = 0.92).

#### 3.1.3. Association Between the p.C282Y rs1800562 Polymorphism and Abdominal Obesity

Based on the random effects model, the *HFE* p.C282Y polymorphism was not significantly associated with the risk of abdominal obesity. No significant differences were observed between any carrier group and non-carriers (p.C282Y homozygous model 95% CI: 0.53 [0.11, 2.57], *p* = 0.43).

#### 3.1.4. Association Between the p.C282Y (rs1800562) Polymorphism and Serum HDL Cholesterol Levels

Based on the random effects model, the *HFE* p.C282Y polymorphism was not significantly associated with serum HDL concentration. No significant differences were observed between any carrier group and non-carriers, regardless of genotype (p.C282Y homozygous model 95% CI: 0.10 [−0.02, 0.22], *p* = 0.10; heterozygous model 95% CI: −0.01 [−0.21, 0,20], *p* = 0.96).

#### 3.1.5. Association Between the p.C282Y rs1800562 Polymorphism and Serum Triglyceride Levels

Based on the random effects model, the *HFE* p.C282Y polymorphism was not significantly associated with serum triglyceride levels. No significant differences were observed between any carrier group and non-carriers, regardless of genotype (p.C282Y heterozygous model 95% CI: 0.04 [−0.56, 0.64], *p* = 0.90; homozygous model 95% CI: −0.08 [−0.70, 0,55], *p* = 0.81).

### 3.2. Risk of Bias Assessment

**Figure 4:**
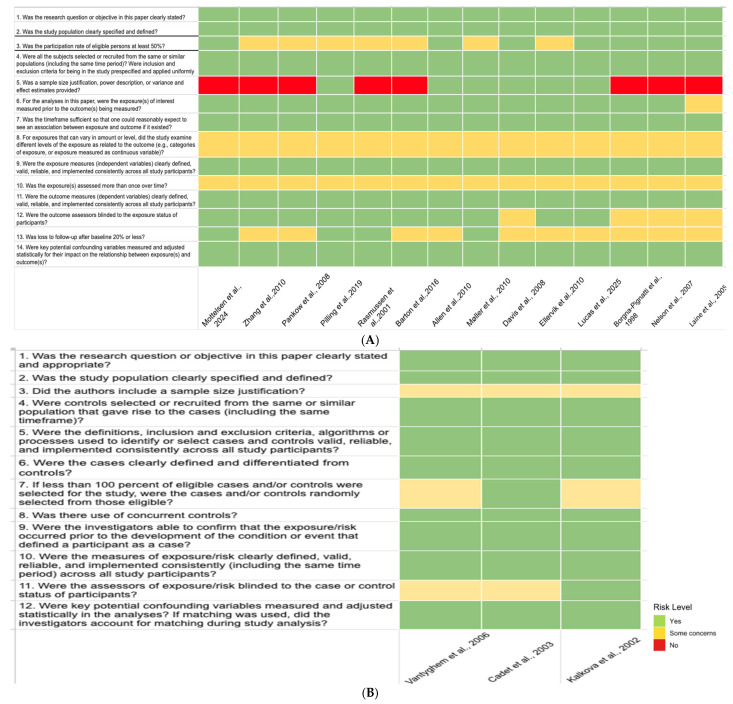
Observational cohort and cross-sectional studies were assessed using the NIH Quality Assessment Tool for Observational Cohort and Cross-Sectional Studies (**A**), whereas case–control studies were assessed using the NIH Quality Assessment Tool for Case–Control Studies (**B**).

The results of the risk of bias assessment are presented in Figure 4. Overall, several common methodological limitations were identified across the included studies, most notably the lack of sample size justification, limited assessment of exposure levels, and absence of repeated exposure measurements over time.

Color palettes were customized to represent low (some concerns) and high risk in a visually accessible manner, using calm tones for clarity.

### 3.3. Publication Bias

**Figure 5:**
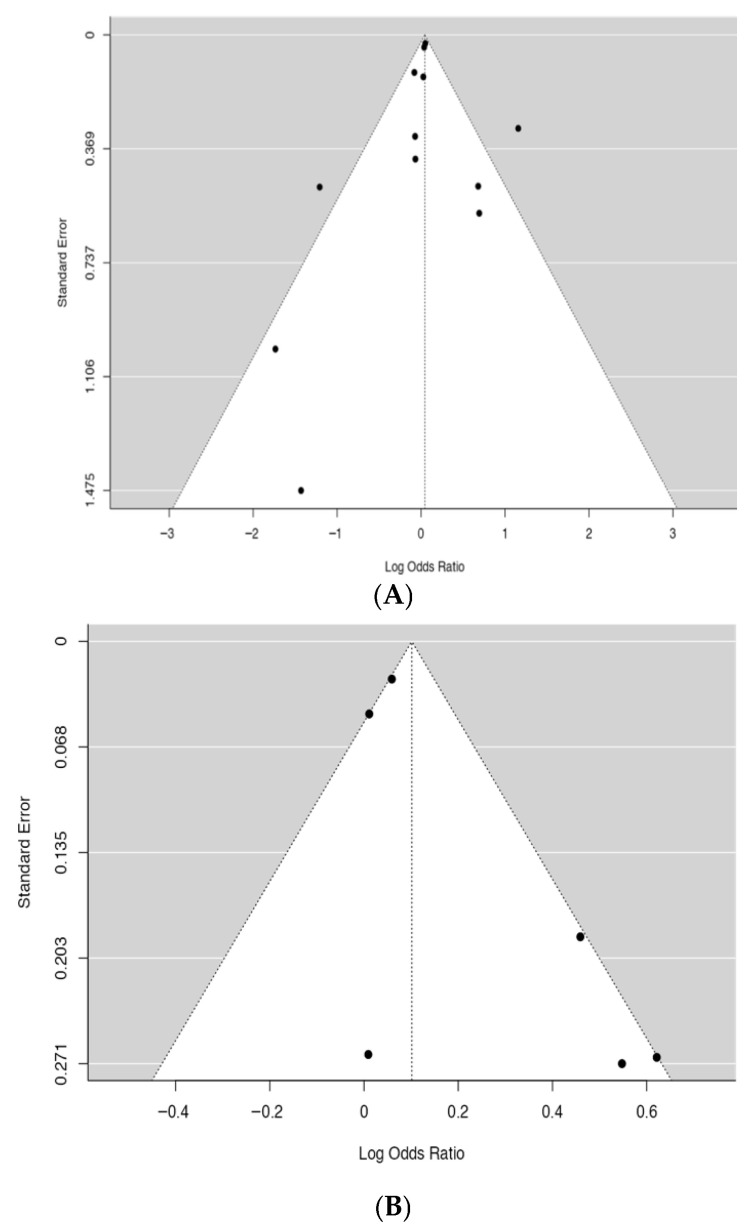
Funnel plots for the relationships between the p.C282Y (rs1800562) polymorphism and (**A**) diabetes or (**B**) hypertension.

Funnel plots (Figure 5) revealed a largely symmetrical distribution of studies, indicating no evidence of publication bias. Publication bias was further assessed using Egger’s regression test, which indicated no significant publication bias for the associations between the *HFE* p.C282Y (rs1800562) polymorphism and diabetes (allele contrast model: Egger’s test, *p* = 0.180) or hypertension (Egger’s test, *p* = 0.398).

## 4. Discussion

To the best of our knowledge, this is the first meta-analysis to comprehensively evaluate the association between the p.C282Y SNP and multiple components of metabolic syndrome within a single analytical framework. Based on the available data, the *HFE* p.C282Y polymorphism does not demonstrate a stable association with the components of metabolic syndrome and, thus, cannot be considered as an independent marker of metabolic risk.

### 4.1. HFE p.C282Y Polymorphism and Diabetes

We did not find a statistically significant association between the *HFE* p.C282Y variant and diabetes under the codominant model. No gene–dose effect was observed, and neither the homozygous nor heterozygous models demonstrated a significant increase in diabetes risk. Previous meta-analyses have evaluated the association between *HFE* variants and type 2 diabetes, including the study by Rong et al., which reported a modest association between the p.H63D variant and type 2 diabetes risk, whereas no significant association was observed for p.C282Y [31]. These findings suggest that the contribution of different *HFE* variants to metabolic risk may vary and underscore the importance of variant-specific evaluation. However, according to the results of the literature review, extensive population-based cohort studies provide evidence that homozygous patients with *HFE* p.C282Y have a significantly elevated risk for diabetes, independent of overt iron overload or abnormal iron indices at baseline [19]. In individuals with hereditary hemochromatosis, the prevalence of diabetes ranges from 13% to 25%, with its pathogenesis involving both β-cell injury and hepatic insulin resistance [32]. In patients with congenital hemolytic anemias and iron overload, prolonged exposure to elevated ferritin is associated with a nearly threefold increased odds of developing diabetes [7]. The pathogenesis of diabetes in *HFE* p.C282Y homozygotes is multifactorial; while pancreatic iron deposition (siderosis) can contribute to β-cell dysfunction, other factors such as insulin resistance, increased body mass index, family history of diabetes, and the presence of cirrhosis also play important roles [33]. Notably, while reflecting body iron stores, serum ferritin does not independently predict the risk of diabetes in this population, and phlebotomy to reduce iron stores does not consistently improve glycemic control in affected individuals [34]. Associations reported in other studies may be partly attributable to the genetic complexity of the 6p region, which is characterized by multiple susceptibility loci and extensive linkage disequilibrium. This underscores the importance of considering the broader genomic context when interpreting association studies involving variants located within this locus [35].

### 4.2. HFE p.C282Y Polymorphism and Hypertension

No significant association with hypertension was observed under the heterozygous and homozygous models, which may be due to the low frequency of p.C282Y homozygosity and the resulting limited statistical power. The current consensus in the medical literature is that p.C282Y homozygosity is primarily associated with hepatic, endocrine, and musculoskeletal complications, and not with hypertension as a direct clinical manifestation. Simple heterozygosity for p.C282Y or compound heterozygosity with p.H63D does not result in clinically significant iron overload or increased risk of hypertension [36]. However, iron overload is closely linked to metabolic syndrome, of which essential hypertension is a cardinal component. Epidemiological studies also support an association between excess iron and increased risk of cardiovascular disease, including hypertension, likely mediated by oxidative stress and endothelial dysfunction [4].

### 4.3. HFE p.C282Y Polymorphism and Abdominal Obesity

We found no evidence for an association between *HFE* p.C282Y and abdominal obesity, as the pooled estimates were not statistically significant. The literature does not demonstrate a consistent association between the p.C282Y mutation and other metabolic syndrome components, and the relationship between the *HFE* p.C282Y mutation and abdominal obesity remains nuanced. Large population-based studies have indicated that individuals homozygous for the *HFE* p.C282Y mutation do not have a higher prevalence of abdominal obesity, when compared with those without *HFE* mutations. Specifically, mean waist circumference and the prevalence of obesity are similar or slightly lower in p.C282Y homozygotes compared with controls, with no significant differences observed in either men or women. Overall, the p.C282Y mutation appears to be associated with an increased risk of diabetes but not with increased abdominal adiposity, while its relationships with other components of metabolic syndrome remains less clear [37].

### 4.4. HFE p.C282Y Polymorphism and Dyslipidemia (Level of HDL and Triglyceride)

In our meta-analysis, the associations between the p.C282Y polymorphism and HDL and triglyceride levels were not statistically significant, with no observed differences between carriers of the polymorphism and individuals with the wild-type genotype. Overall, while the p.C282Y mutation is associated with altered iron metabolism and reduced LDL cholesterol, the available clinical evidence does not support significant or consistent relationships between the p.C282Y mutation and HDL cholesterol and triglyceride levels in humans [16].

### 4.5. Strength and Limitations

The validity of this systematic review and meta-analysis indicates that the results obtained generally reflect the existing body of data on the issue under study; however, they should still be interpreted with caution. Most of the included studies were conducted many years ago and mainly in populations of European origin, which ensures a certain genetic uniformity but, at the same time, limits the possibility of generalizing the results to other ethnic groups.

The total number of included studies remained relatively small, which reduces the statistical power and increases the uncertainty of the combined effect estimates. It should also be noted that in many studies, the number of carriers of the homozygous p.C282Y variant was extremely limited, which significantly reduced the reliability of effect estimates for this genetic model and increased the width of the confidence intervals. The inclusion of English-language studies only may have introduced language bias and potentially affected the generalizability of the findings.

The main sources of methodological heterogeneity were differences in research design, sources of control group formation (population samples or hospital controls), diagnostic criteria, and sample sizes, which ranged from small studies to large population cohorts. Many studies had insufficient capacity to identify moderate genetic effects, which could lead to false negative results at the individual study level; in contrast, the meta-analytical approach allowed for the aggregation of data, thus increasing the overall statistical reliability of estimates.

Additional factors potentially affecting internal validity include incomplete reporting of compliance with the Hardy–Weinberg equilibrium, variability in genotyping methods, and differences in quality control procedures for genetic data. The degree of statistical adjustment for significant confounders (age, gender, body mass index, and other metabolic parameters) varied significantly between studies, with several studies using unadjusted estimates.

A separate limitation is the analysis of diabetes—in several studies, no distinction was made between the different phenotypes of the disease, and data on type 1, type 2, and gestational diabetes were included in the meta-analysis. Such phenotypic heterogeneity could affect the combined effect estimates and contribute to inter-study variability.

Deviations from the Hardy–Weinberg equilibrium reported in some control groups may have introduced potential bias and should be considered when interpreting the findings of this meta-analysis.

Despite these limitations, the systematic literature search, transparent inclusion criteria, and relative consistency of results across larger population cohorts support the validity of the present review and meta-analysis. In general, the data obtained represent a reasonable synthesis of the available evidence regarding the role of the p.C282Y polymorphism in the formation of components of metabolic syndrome and emphasize the need for further well-planned, large-scale, and ethnically diverse studies to better understand the involvement of the p.C282Y variant of the *HFE* gene in the pathogenesis of individual manifestations of metabolic syndrome. The present meta-analysis did not provide convincing evidence for any association between the *HFE* p.C282Y polymorphism and the considered components of metabolic syndrome. These findings suggest that this variant cannot be considered as an independent genetic marker of metabolic risk, based on current evidence. The high heterogeneity between studies may reflect population differences, design variability, and possible gene–environment interactions. Further large, methodologically homogeneous studies are needed to clarify potential population-specific effects.

## 5. Conclusions

Taken together, the available data did not allow us to confirm the presence of any statistically significant association between the *HFE* p.C282Y polymorphism and the components of metabolic syndrome.

## Figures and Tables

**Table 1:** The characteristics of eligible qualified studies.

First Author, Year	Race	Source of Controls	Sample Size		*p*-Value for HWE	Adjustment Factors	Genotyping Methods
			Cases	Controls			
Diabetes							
1. Pankow et al., 2008 [16]	Caucasian	Population	1126	6768	*p* > 0.05	Not adjusted	TaqMan method
2. Cadet et al., 2003 [17]	Caucasian	Population	94	655	NR	Age and sex	NR
3. Nelson et al., 2007 [18]	Caucasian	Hospital	18	75	NR	Age, sex, ethnicity, BMI	NR
4. Pilling et al., 2019 [19]	Caucasian	Population	67,304	383,909	*p* > 0.05	Age, assessment center	Genotyping microarray
5. Vantyghem et al., 2006 [20]	Caucasian	Hospital	77	39	NR	Not adjusted	PCR
6. Zhang et al., 2010 [21]	Caucasian	Population	72	409	*p* = 0.36	Age, time elapsed from baseline	PCR
7. Barton et al., 2016 [22]	Caucasian	Population	223	449	NR	Age, sex, BMI	PCR
8. Rasmussen et al., 2001 [23]	Caucasian	Population	65	713	NR	Age, race, gender	PCR
9. Borgna-Pignatti et al., 1998 [24]	Caucasian	Hospital	8	208	NR	Not adjusted	PCR
10. Kanková et al., 2002 [25]	Caucasian	Population	27	299	*p* < 0.05	Not adjusted	NR
Hypertension							
1. Ellervik et al., 2010 [26]	Caucasian	Population	3565	24,064	Reported, not stated	Age, gender, BMI	TaqMan assay
2. Møller et al., 2010 [27]	Caucasian	Hospital	73	594	*p* = 0.922	Not adjusted	PCR
3. Rasmussen et al., 2001 [23]	Caucasian	Population	65	713		Age, race, gender	PCR
4. Davis et al., 2008 [28]	Caucasian	Population	139	1092	Reported; *p*-value not stated	Age, diabetes duration	PCR
5. Zhang et al., 2010 [21]	Caucasian	Population	72	409	*p* = 0.36	Age, time elapsed from baseline	PCR
6. Mottelson et al., 2024 [15]	Caucasian	Population	12,936	87,313	*p* < 0.05	Age and sex	TaqMan/KASPar KASPar
Abdominal obesity							
1. Lucas et al., 2025 [29]	Caucasian	Population	2899	268,007	NR	Alcohol intake, smoking status, education, Townsend deprivation index, physical activity, and any baseline diagnosis of viral hepatitis alcoholic liver disease	WES
2. Lainé et al., 2005 [30]	Caucasian	Population	47	31	NR	Sex (gender), BMI	NR
HDL							
1. Pankow et al., 2008 [16]	Caucasian	Population	1126	6768	*p* > 0.05	Not adjusted	TaqMan method
2. Davis et al., 2008 [28]	Caucasian	Population	139	1092	Reported; *p*-value not stated	Age, diabetes duration	PCR
Triglyceride							
1. Pankow et al., 2008 [16]	Caucasian	Population	1126	6768	*p* > 0.05	Not adjusted	TaqMan method
2. Davis et al., 2008 [28]	Caucasian	Population	139	1092	Reported; *p*-value not stated	Age, diabetes duration	PCR

BMI, body mass index; HWE, Hardy–Weinberg equilibrium; HDL, high-density lipoprotein; WES, whole exome sequencing; PCR, polymerase chain reaction; NR, not reported.

## Data Availability

The data supporting the findings of this study are derived from previously published studies, which are cited in the article. All data used for the meta-analysis were extracted from these sources, and no new primary data were generated. Further details are available from the corresponding author upon reasonable request.

## Supplementary Materials

The following supporting information can be downloaded at: https://www.mdpi.com/article/10.3390/medicina62030589/s1, Table S1: PRISMA checklist; Table S2: Search strategy for the databases.
